# Determinants of outpatient substance use disorder treatment length-of-stay and completion: the case of a treatment program in the southeast U.S

**DOI:** 10.1038/s41598-023-41350-8

**Published:** 2023-08-26

**Authors:** Aaron Baird, Yichen Cheng, Yusen Xia

**Affiliations:** Institute for Insight, Robinson College of Business, Georgia State University, 55 Park Place, Atlanta, GA 30303 USA

**Keywords:** Health care, Health services

## Abstract

Successful outcomes of outpatient substance use disorder treatment result from many factors for clients—including intersections between individual characteristics, choices made, and social determinants. However, prioritizing which of these and in what combination, to address and provide support for remains an open and complex question. Therefore, we ask: What factors are associated with outpatient substance use disorder clients remaining in treatment for > 90 days and successfully completing treatment? To answer this question, we apply a virtual twins machine learning (ML) model to de-identified data for a census of clients who received outpatient substance use disorder treatment services from 2018 to 2021 from one treatment program in the Southeast U.S. We find that primary predictors of outcome success are: (1) attending self-help groups while in treatment, and (2) setting goals for treatment. Secondary predictors are: (1) being linked to a primary care provider (PCP) during treatment, (2) being linked to supplemental nutrition assistance program (SNAP), and (3) attending 6 or more self-help group sessions during treatment. These findings can help treatment programs guide client choice making and help set priorities for social determinant support. Further, the ML method applied can explain intersections between individual and social predictors, as well as outcome heterogeneity associated with subgroup differences.

## Introduction

Outpatient programs can be effective for substance use disorder (SUD) treatment, particularly when detoxification is not needed (or has been completed) and continuous monitoring is not (or is no longer) needed^1^. Such treatment is most often successful if clients remain in a program for more than 90 days^2^ and if the program is not exited “against medical advice” or earlier than recommended due to administrative reasons, such as due to extended incarceration^2^. Yet, we also know there can be substantial variation in treatment completion due to individual differences (e.g., race, age, drug of choice)^3^ and social differences (e.g., employment and housing disruption)^4^. Thus, research is needed on which combinations (i.e., ‘intersections,’ as described more below) of factors most often lead to positive outcomes.

What is not as well-known is the specific situations under which clients are most likely to remain in and successfully complete outpatient SUD treatment. While guidelines exist^5,6^, many such guidelines focus on medical and/or mental health management and do not evaluate impacts on outcomes associated with interactions between individual and social characteristics. To address this challenge, emerging work has started to identify nuanced conditions associated with treatment success, such as machine learning (ML) based work which has found that recent frequency of substance use and self-motivation are significant predictors of treatment completion^7^ as well as work that has identified general disparities associated with treatment completion^3^. Further, some SUD outpatient providers, such as the one that is the source of the data in this study, now focus on treating the “whole person” (i.e., individual as well as social and support needs) rather than only symptoms or issues associated directly with substance use. However, we do not yet sufficiently understand how individual and social characteristics interact when predicting treatment completion. Further, especially for priority assistance populations, including minority populations, not enough is known about what factors are most beneficial in treatment completion.

To address open questions regarding how individual and social factors interact toward affecting treatment success, we situate this study in the domain of intersectionality theory^8–10^. Intersectionality theory focuses on how determinants, and especially demographic and social determinants, interact when evaluating causes of effects. This body of work assumes that determinants often intertwine in impacting effects as opposed to having individual, isolated impacts^11^. Thus, we apply and contribute to this theory in two ways: (1) by identifying intersectionality between individual and social determinants in the SUD outpatient treatment context of study, and (2) by furthering the application of the virtual twins method to identifying true intersections between variables^10^, rather than additive or stepwise approaches noted as not being ideal for identifying intersections^9^.

Thus, we conducted a study, in partnership with an outpatient SUD treatment program, to determine what factors, and in what combination, most impact preferred outcomes. The population of clients was primarily African American males, residing in the Southeastern U.S. We applied ML—specifically, virtual twins^12^, which includes prediction, feature importance, and classification tree-based subgroup analysis—to determine which factors, in what combination, and at what levels were most predictive of longer lengths-of-stay in treatment (> 90 days) and successful treatment completion (did not leave against medical advice or for other reasons, such as extended incarceration).

Ultimately, we find that for this population, the best predictors of treatment success are the number of self-help groups attended during treatment, followed by the number of goals set by the client. When analyzing the subgroups of clients most likely to attend at least one self-help group and those most likely to set one or more goals, we find that access to primary care provider (PCP) and to the supplemental nutrition assistance program (SNAP) can enhance the probability of engaging in these beneficial activities (i.e., self-help group attendance; goal setting). We also find several additional subgroup nuances, reported later. These findings can be used by SUD treatment providers to determine how to best prioritize the myriad of client guidance and support recommendations (or services) available.

## Methods

### Research design

This observational prognostic and quality improvement study was conducted between August 2022 and May 2023 using de-identified client data from 2018 to 2021 from an outpatient SUD treatment program in the Southeastern region of the U.S. The Georgia State University (GSU) Institutional Review Board (IRB) approved the study as exempt due to the data being de-identified, thus not requiring informed consent (IRB Number: H22253, Reference Number: 367768). All methods were performed in accordance with relevant guidelines and regulations. The study followed the Transparent Reporting of a Multivariable Prediction Model for Individual Prognosis or Diagnosis (TRIPOD) guidelines^13^, as the first stage is a predictive model. The study also followed the Standards for Quality Improvement Reporting Excellence (SQUIRE) guidelines^14^, as the second stage of the method applies a classification tree toward more fully understanding impacts on outcomes.

### Data source

De-identified data for a census of clients (N = 256) who utilized outpatient SUD treatment services from 2018 to 2021 provided by one SUD treatment program in Southeast region of U.S. for the purposes of better understanding the predictors of longer stays (> 90 days) and successful treatment completion (stayed until treatment was deemed completed by clinicians; did not leave against medical advice or for other reasons). Data collection required examination of both electronic and paper records as well as chart reviews. Final data also lagged by a number of months as charts and paperwork were being completed, which is why the data is greater than one year old.

We also note that many studies in this domain make use of the Treatment Episode Data Set (TEDS) available from the Substance Abuse and Mental Health Services Administration (SAMHSA)^3,15,16^. While these data have breadth advantages, especially in that they contain many millions of rows representing admissions or discharges throughout the country, they lack detailed variables in regard to client choices and social determinants. Therefore, while we acknowledge our sample is limited to a specific region and client population, from a single provider of treatment services, our dataset is unique with the advantage of including detailed client choices and social determinants. Thus, the study reported here is unique in that it applied an emerging ML method (virtual twins) to a narrow sample (N = 256) with the benefit of more specific variables than used in other studies of SUD treatment.

### Target variables

For the outcomes considered in this study, we used two target variables consistent with the dependent (or target) variables used in SUD treatment research: (1) > 90 days length-of-stay (coded as 0 if <  = 90 days and 1 if > 90 days), and (2) successful treatment completion (coded as 0 if left against medical advice or for any administrative reason, such as extended incarceration, and 1 if discharged as successfully completed treatment)^2,17^. We note that we asked the treatment provider if any of the discharges coded as zero (i.e., not completed successfully) were for reasons such as moving, death, transfer, or incarceration. They responded that none of their clients had ever moved out of the area while receiving treatment or died during treatment. A very small percentage needed to be transferred to a higher-level facility or were administratively discharged due to extended incarceration. Thus, nearly all the discharges coded as zero (i.e., not completed successfully) were due to leaving against medical advice.

We further note that we chose to use a dichotomous variable as the first stage dependent (target) variable for three reasons: (1) the SUD treatment provider we were working with was interested in factors that contributed to > 90 days length of stay, which is one of their metrics for success and is also substantiated in the literature (i.e., > 90 days of treatment often leads to better outcomes than < 90 days)^2^, (2) the applications of virtual twins in the literature within similar research contexts have also used a dichotomous variable in the first stage^10^, and (3) classification, as opposed to regression, was the best fit in our view for the first stage as the true output of the models reported here are second stage decision trees. Attempting to predict a precise LOS (i.e., not dichotomized) seemed less applicable than classification as specified by the facility.

### Predictor variables

42 variables were provided that include substance use information and history (e.g., primary and secondary substance of use, prior use of substances), demographics (e.g., race, gender, age, marital status, education), medical details and history (e.g., mental health diagnosis if any, on prescription medication, linked to a primary care provider), social determinants (e.g., access to transportation, linked to SNAP, employment/income), client choices (e.g., number of goals set, number of self-help groups attended), other relevant information (e.g., referral source, criminal system involvement at admission), and outcomes (length of stay, discharge status). Of the 42 variables provided, 2 were target variables (> 90 days length of stay; discharge status), leaving 40 variables for use in prediction. All 40 variables were included in the prediction models. (See Supplements 1 and 2 for descriptive statistics that include full variable lists).

Two of the variables, setting of goals and attending self-help groups, are explained more here as they become important in the results.

Regarding the setting of goals, goals were always set face-to-face with a counselor. Individuals were supported through developing Specific, Measurable, Achievable, Relevant, and Timely (SMART) goals utilizing a worksheet, which required identifying, documenting, and at times revising, a stated goal to ensure it was SMART. This process also included discussion and documenting why each goal was important to the individual, benefits of achieving the goal, potential obstacles and solutions, specific action steps, as well as, identifying people who would be supportive and helpful in the process of working toward goals. Individuals typically created goals based on the following domains: psychiatric (which includes substance use/abuse and mental health goals), housing/living, family/social, and work/education.

Regarding self-help group attendance, Alcoholics Anonymous (AA) and Narcotics Anonymous (NA), which are designed to help individuals recovering from substance abuse and addiction, are the groups that individuals are required to participate in as a part of this treatment program. In addition to those two groups, some individuals also participated in a Christian-based recovery program designed to help individuals struggling with a wide range of hurts, habits, and hang-ups, including but not limited to addiction, trauma, grief, anxiety, and relationship issues.

### Machine learning approach applied: virtual twins

Developing prediction models is helpful for understanding who is most likely to remain in treatment > 90 days or successfully complete treatment, but prediction models are often opaque as the goal is often high accuracy rather than explanation and transparency^15^. One way to open the black box of prediction models is to apply subgroup analysis, particularly with classification trees. However, it is also well known that classification trees are prone to overfitting^18^. To address the dual challenge of explaining why certain outcomes are more likely while also avoiding overfitting to the extent possible, new ML methods have been developed^19^, including the “virtual twins” method^3,10,12^.

The virtual twins method is based on use of counterfactuals where there are two possibilities, but only one can be observed. The method is designed to first estimate the difference in probability between the observed and the counterfactual (i.e., ${Z}_{i}={P}_{1i}-{P}_{0i}$), where for client *i* $, {P}_{1i}$ represents being in the treated group (i.e., in the subgroup) and ${P}_{0i}$ represents being in the control group (i.e., not in the subgroup). Then, in the second stage, the method is designed to determine what variables cause this probability difference, by modeling the impact of the covariates (predictors) ${X}_{i}$ on the probability difference ${Z}_{i}$.

Specifically, in the first stage, a probability is determined for an outcome for every observation where the subgroup variable represents what was observed. Then, to establish a counterfactual or a “virtual twin,” a second probability is calculated for every observation with the subgroup variable (i.e., whether goals were set by the client; whether self-help groups were attended by the client) switched to the opposite (i.e., for those with at least one goal set, it is switched to 0 and vice versa; same process for self-help group attendance). The difference of these two probabilities is then calculated per observation (e.g., P(one or more goals set) – P(no goals set), or P(one or more self-help groups attended) – P(no self-help groups attended). This difference becomes the target variable in the second stage. In the second stage, we applied a classification tree to determine which factors, which are the same predictor variables in the first stage other than the subgroup variable, cause the probability difference^12,20^.

## Results

### Client characteristics

Most of those in this sample were Black or African American (87.9%), aged 41–60 (70.7%), single (85.2%), had completed grade 12 or higher (74.7%), primarily used alcohol (37.9%) or cocaine (43.4%), were court ordered to participate (80.5%) or had criminal system involvement at the time of admission (52.3%), were not receiving SNAP at intake (77.3%), already had a primary care provider (PCP) at intake (78.1%), were linked to a PCP during treatment (68.4%), and did not have a secondary mental health diagnosis (71.9%). Regarding outcomes, 57.8% stayed longer than 90 days prior to discharge and 44.9% were discharged on the advice of clinicians (i.e., successfully completed treatment). For details and additional descriptive statistics, please see Supplements 1 and 2.

### Virtual twins: stage 1 performance and results

The first step in this first stage was to develop prediction models for our two target variables (> 90 days; successful treatment completion). For both target variables, logistic regression, gradient boosting, random forest, and deep learning methods were applied to prediction. In every application of each of these methods (i.e., logistic regression, gradient boosting, random forest, and deep learning), a tenfold cross validation was applied with a 70% training and 30% testing data split.

For the > 90 days target variable, prediction model accuracies ranged from 0.731 (gradient boosting) to 0.751 (logistic regression) and AUCs ranged from 0.748 (deep learning) to 0.782 (random forest). Given the comprehensiveness of the AUC score^21^, we selected the model with the highest AUC score for prediction, which was the random forest model. See Supplement 3 for more details.

We note that AUC scores represent the area under the receiving operator characteristic curve (ROC), which is the area under the curve plotted for all true positive and true negative rates at every classification threshold between zero (0) and one (1) for the model being evaluated. The integral of the ROC curve results in a single number, which represents the area under this curve (i.e., the AUC). The range of AUC is between 0 and 1. The larger the value, the more accurate of the prediction. When it is closer to 1, prediction accuracies (i.e., true positive and true negative rates) are closer to 100% percent accurate. The AUC score has been shown to be a good overall metric for evaluating ML models^21^ and, thus, we used AUC scores as our evaluation metric for prediction accuracy.

For the successful treatment completion target variable, prediction model accuracies ranged from 0.797 (deep learning) to 0.831 (gradient boosting and random forest) and AUC scores ranged from 0.841 (deep learning) to 0.886 (random forest). To be consistent across model selection procedures, we again selected the model with the highest AUC, which coincidently was the random forest model. See Supplement 4 for more details.

**Figure 1:**
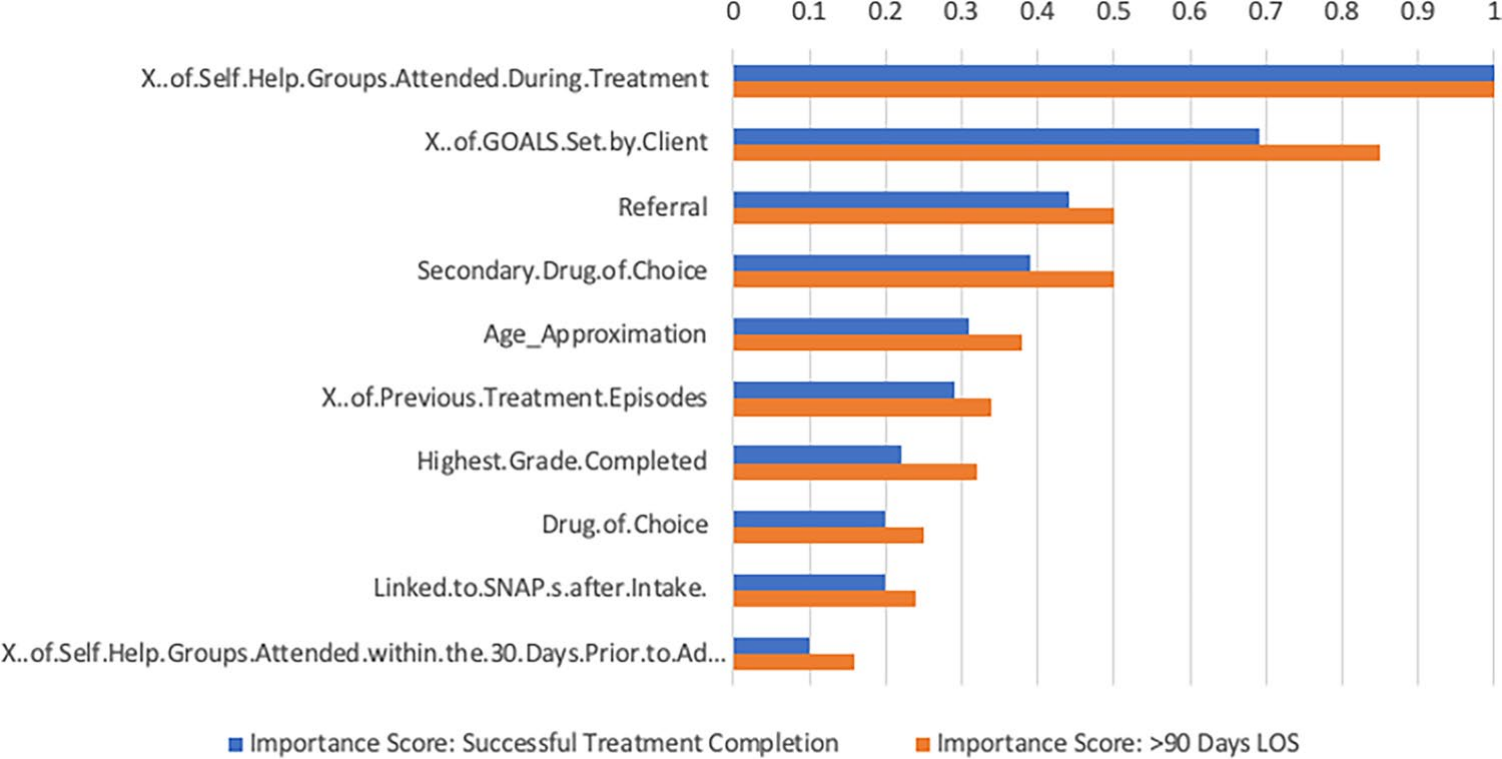
Feature importance for virtual twins stage 1 random forest prediction models. Feature importances are scaled from 0 to 1, with 1 being the most important.

We then ran feature importance for both the random forest prediction models (i.e., for > 90 days and successful treatment completion prediction models). The feature importance method returns a scaled result, from 0 to 1, where 1 is the most important, followed by variables of lesser importance. In both cases, the most important two variables were: (1) the number of self-help groups attended during treatment (scaled score of 1 in both feature importance results), and (2) the number of goals set by the client (scaled score of 0.85 for the > 90 days model and 0.69 for the treatment completion model). See Fig. 1 for details and for the top 10 most important features.

### Virtual twins: stage 2 results

In the second stage, a classification tree was used to determine which combinations of predictor variables (and at what levels of each) had the most (and least) impact on the difference in probabilities for the selected subgroup variable. While some studies applying this method utilize a predetermined subgroup variable (e.g., race^10^) or explore a number of possible subgroups (e.g., not only race, but also co-occurrence of a mental health disorder, income from a job, health insurance access, gender, age, veteran status, and primary substance of choice^3^), we opted to define our subgroups as a result of the feature importance from the first stage. In other words, we sought more explanation of not only which predictors are most important in predicting selected outcomes (> 90 days; successful treatment completion), but also which subgroups were most likely to engage in behaviors beneficial to suggested duration (> 90 days) and completion.

**Figure 2:**
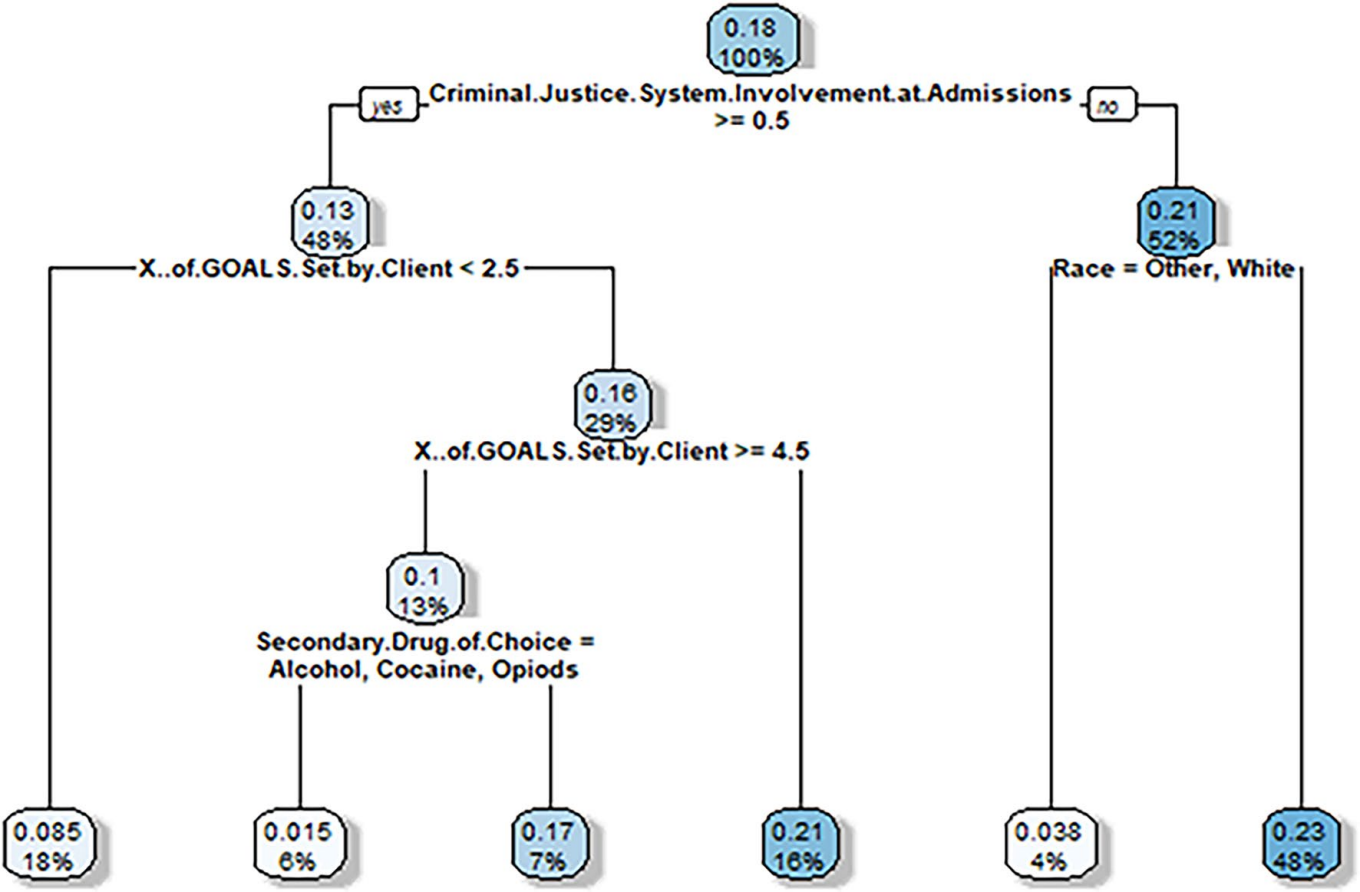
Virtual twins stage 2 classification tree results for clients who stayed in treatment for > 90 days and attended one or more self-help group sessions during treatment. Nodes show two numbers: (1) the top number is increased (or decreased) probability of staying > 90 days when attending 1 or more self-help groups, (2) the bottom number is the percent of sample for which the node applies. Then, branches represent splits in the sample. All left branches represent that the branching condition is met (yes) and right branches represent that the branching condition is not met (no). The hue of each node is darker when the percentage impact (the top number) is higher. Thus, the darkest nodes have the greatest impact and the lightest have the least.

**Figure 3:**
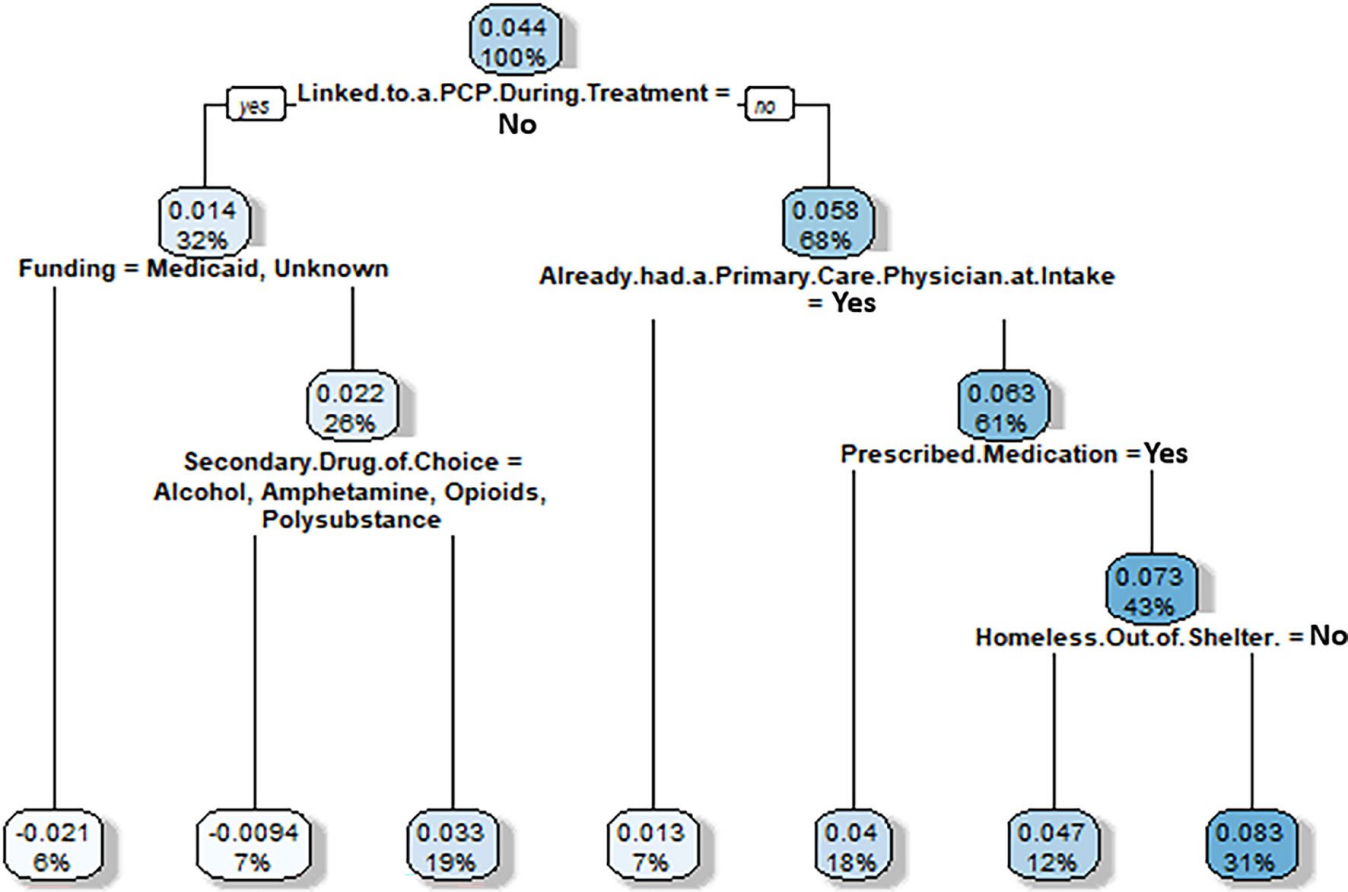
Virtual twins stage 2 classification tree results for clients who stayed in treatment for > 90 days and set one or more treatment goals. Nodes show two numbers: (1) the top number is increased (or decreased) probability of staying > 90 days when setting 1 or more goals, (2) the bottom number is the percent of sample for which the node applies. Then, branches represent splits in the sample. All left branches represent that the branching condition is met (yes) and right branches represent that the branching condition is not met (no). The hue of each node is darker when the percentage impact (the top number) is higher. Thus, the darkest nodes have the greatest impact and the lightest have the least.

**Figure 4:**
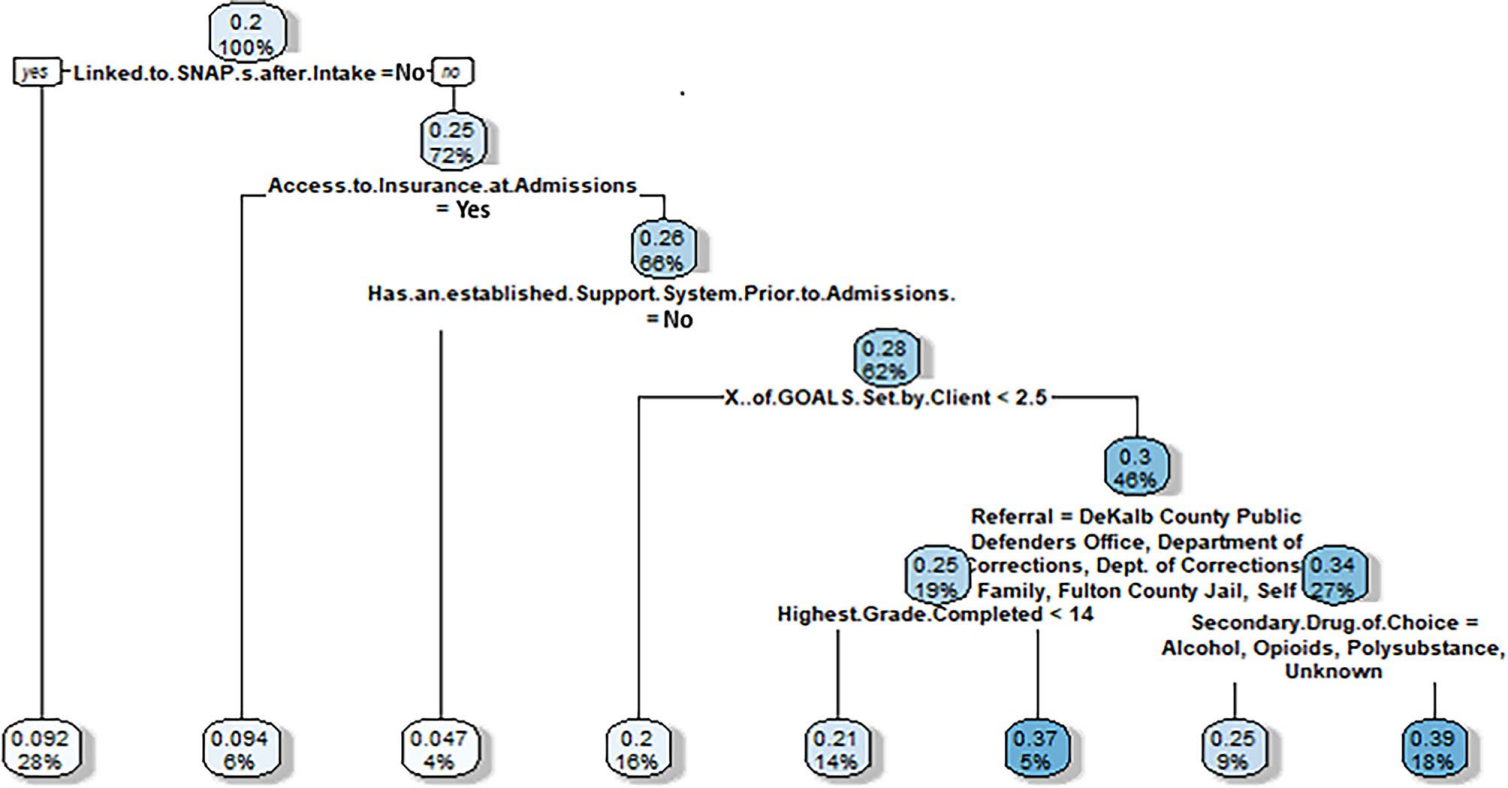
Virtual twins stage 2 classification tree results for clients who successfully completed treatment and attended one or more self-help group sessions during treatment. Nodes show two numbers: (1) the top number is increased (or decreased) probability of completing treatment successfully (i.e., discharged by clinician rather than against medical advice) when attending 1 or more self-help groups, (2) the bottom number is the percent of sample for which the node applies. Then, branches represent splits in the sample. All left branches represent that the branching condition is met (yes) and right branches represent that the branching condition is not met (no). The hue of each node is darker when the percentage impact (the top number) is higher. Thus, the darkest nodes have the greatest impact and the lightest have the least.

**Figure 5:**
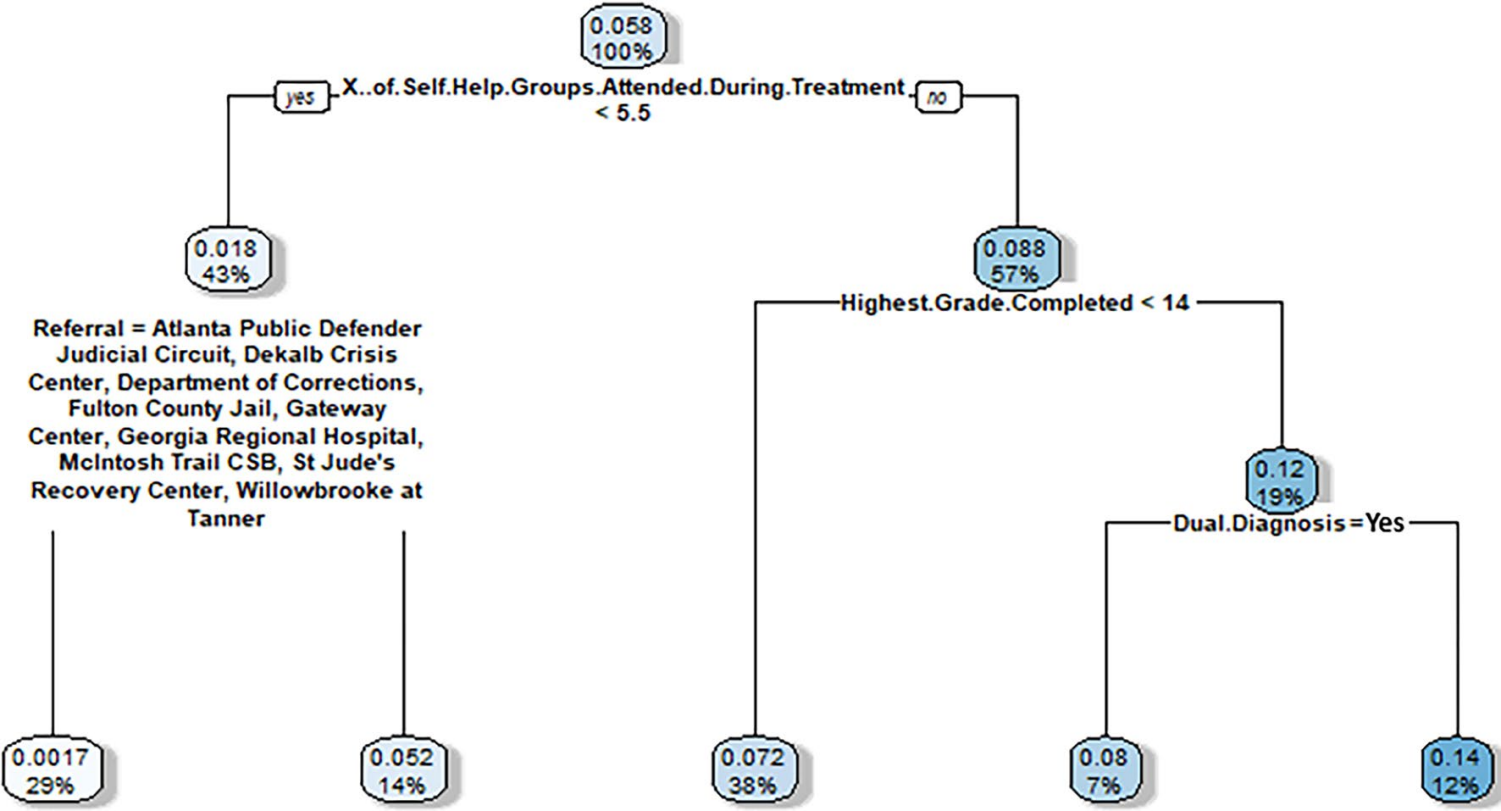
Virtual twins stage 2 classification tree results for clients who successfully completed treatment and set one or more treatment goals. Nodes show two numbers: 1) the top number is increased (or decreased) probability of completing treatment successfully (i.e., discharged by clinician rather than against medical advice) when setting 1 or more goals, (2) the bottom number is the percent of sample for which the node applies. Then, branches represent splits in the sample. All left branches represent that the branching condition is met (yes) and right branches represent that the branching condition is not met (no). The hue of each node is darker when the percentage impact (the top number) is higher. Thus, the darkest nodes have the greatest impact and the lightest have the least.

Thus, we selected the top two most important features from stage 1—number of self-help groups attended; number of goals set— for stage 2 analyses, which happened to be consistent between the two prediction models (i.e., > 90 days; successful treatment completion). Thus, the variables selected for further analyses were: (1) no self-help groups attended (${P}_{0i}$) vs. one or more self-help groups attended (${P}_{1i}$), and (2) no goals set (${P}_{0i}$) vs. one or more goals set (${P}_{1i}$),

We generated two classification trees per prediction model, one for the self-help group analyses and one for the goals set analyses. In other words, for the > 90 days prediction model, classification trees were generated for: (1) self-help subgroup virtual twin probability difference (Fig. 2), and (2) number of goals set virtual twin subgroup probability difference (Fig. 3). Then, for the treatment completion prediction model, classification trees were again generated for the same two subgroups: (1) self-help subgroup virtual twin probability difference (Fig. 4), and (2) number of goals set subgroup virtual twin probability difference (Fig. 5).

#### Results for stayed > 90 days, self help group attendance (0 vs. > 0)

The results show that, when using the > 90 days prediction model, clients most influenced to attend self-help groups were those with no criminal system involvement who were not of other or white race (23% more likely, representing 48% of the sample). This is followed closely by those with criminal system involvement who set between 2.5 and 4.5 goals (21% more likely, representing 16% of the sample).

#### Results for stayed > 90 days; number of goals set (0 vs. > 0)

The results show that, when using the > 90 days prediction model, clients most influenced to set goals were those linked to a PCP during treatment (i.e., the “no” branch is where linked to a PCP is not equal to “No”, or not false), were not on prescribed medication, and were out of a homeless shelter (8.3% more likely; representing 31% of the sample). We note that these results can be confusing to interpret due to the values of some variables (Yes or No) and the branching conditions (Yes or No). We also note that this tree is relatively deep, leading to specific and perhaps not generalizable results. One way to address this is to evaluate the nodes at higher levels of the tree (i.e., closer to the root node), rather than the final leaves (i.e., nodes at the bottom). In doing this, we note that a client is 5.8% more likely to set at least one goal if they are linked to a PCP during treatment (representing 68% of the sample). Thus, an important and generalizable takeaway is that having a PCP is a beneficial condition.

#### Results for discharge = successfully completed; self help group attendance (0 vs. > 0)

We again focus here on the main takeaways rather than the complexity of the bottom, leaf nodes. The results show that, when using the treatment completion prediction model, clients most influenced to attend one or more self-help groups were those linked to SNAP, who did not have health insurance at admission, who had an established support system, and had set more than 2.5 goals (30% more likely; representing 46% of the sample).

#### Results for discharge = successfully completed; number of goals set (0 vs. > 0)

The results show that, when using the treatment completion prediction model, clients most influenced to set one or more goals were those who attended 5.5 or more self-help groups during treatment, completed grade 14 (some college) or higher, and did not have a dual diagnosis including a mental health disease (14% more likely; representing 12% of the sample).

## Discussion

To better understand the intersecting factors^8,9,11,22^ associated with staying in outpatient SUD treatment for > 90 days as well as factors associated with treatment completion success, we applied a virtual twins ML method to analyze discharges of 256 clients from one treatment program in the Southeast region of the U.S. We first found that attending one or more self-help groups and setting one or more goals are most predictive of success, both for staying > 90 days and for completing treatment successfully. We subsequently analyzed the factors contributing to the probability difference between observed and counterfactual for each outcome (> 90 days; successful treatment completion) and the two most important features (self-help group attendance; setting of 1 or more goals). While there are several nuances in the classification tree findings, the primary results are as follows. For variables over which treatment programs can influence (i.e., variables such as client criminal system involvement are good to know about, but cannot be changed or impacted by treatment programs): (1) being linked to PCP helps clients attend self-help groups more often and thus stay in the program > 90 days), (2) being linked to SNAP helps clients attend more self-help groups and is associated with a higher probability of completing treatment successfully, and (3) attending 6 or more self-help group sessions is associated with setting 1 or more goals, which ultimately increases the probability of clients successfully completing treatment. Thus, overall, primary predictors of success are: (1) attending self-help groups, and (2) setting goals (ideally, > 2.5 goals). Secondary predictors are: (1) being linked to a PCP, (2) being linked to SNAP, and (3) attending 6 or more self-help group sessions.

Prior research on substance use disorder treatment completion has found that factors such as being employed, attending support groups, being court ordered to attend, and have fewer adverse childhood experiences are primary factors in predicting treatment completion^23^; that higher education and more mental health needs are also primary determinants^24^; and that factors such as age, race, and drug of choice can moderate such effects^3,10,25^. What is missing is a more in-depth analysis of the intersections of individual and social determinants, particularly using data and methods well suited to identifying heterogeneity within and between individual and social variables^9^. Further, prior applications of the virtual twins method have focused on prescribed characteristics (i.e., race^10^), but have not yet let feature importance drive which factors are considered in the virtual twins models. Therefore, we contribute by evaluating such individual and social intersections, for two target variables identified via feature importance as the top drivers of success (> 90 days LOS; treatment completion), using the virtual twins ML method. We believe our approach can further be applied to additional client populations, receiving treatment from other providers in other regions, to better understand how such intersections vary by provider, region, and client circumstances. In particular, when seeking to treat the “whole person,” prioritization is needed when considering the many individual and social factors that could be considered. Thus, this study can help providers to either apply the same method to their data to understand what their priorities may be and/or, if their client population is similar, to apply the priorities identified here when providing treatment.

In regard to limitations, while this study has the benefit of high quality, in-depth data, with several variables that represent client choices (e.g., number of self-help groups attended; number of goals set) and social determinants (e.g., PCP linkage, SNAP linkage, access to transportation, social support system, etc.), this study is a sample from a specific treatment program (i.e., one provider of outpatient services) in one geographic location. The results reported here are representative of the highest probability of success for this sample, but more research is needed to determine if similar probability increases can be found in other samples and populations. Especially promising is the application of ML toward better understanding of intersections, which is an exciting opportunity for future research in this area.

## Conclusion

This study identified primary and secondary factors associated with length-of-stay and treatment success. The findings suggest that client choices, social determinants, and access to primary care all play a role in treatment success. We contribute to the overall literature on mental health trends and quality improvement^26,27^.

### Supplementary Information

Supplementary Information.

## Data Availability

Data is available upon request from the corresponding author for the purpose of verifying the results in this study.
