# Supplementary material for: Determinants of outpatient substance use disorder treatment length-of-stay and completion: the case of a treatment program in the southeast U.S
Source: Sci Rep. 2023 Aug 26;13:13961. doi: 10.1038/s41598-023-41350-8 (PMC10460408; doi:10.1038/s41598-023-41350-8)
Supplement: Supplementary file 1 — Supplementary Information. [file 41598_2023_41350_MOESM1_ESM.pdf]

Determinants of Outpatient Substance Use Disorder Treatment Length-of-Stay and Completion: The Case of a Treatment Program in the Southeast U.S.

Aaron Baird[1*]; Yichen Cheng[2]; Yusen Xia[3]

[1]Associate Professor (*corresponding author), Ph.D., Institute for Insight; Robinson College of Business, Georgia State University, 55 Park Place, Atlanta, GA 30303, U.S.; abaird@gsu.edu; 404-413-7639; https://robinson.gsu.edu/profile/aaron-m-baird/

[2] Associate Professor, Ph.D., Institute for Insight; Robinson College of Business, Georgia State University, 55 Park Place, Atlanta, GA 30303, U.S.

[3] Bradford and Patricia Ferrer Professorship in Analytics and Director, Institute for Insight; Ph.D., Robinson College of Business, Georgia State University, 55 Park Place, Atlanta, GA 30303, U.S.

Supplemental information is available as follows:

Supplement 1: Descriptive statistics for categorical variables

Supplement 2: Descriptive statistics for continuous variables

Supplement 3: Virtual twins stage 1 accuracies and AUCs for “>90 days” predictive models

Supplement 4: Virtual twins stage 1 accuracies and AUCs for “successfully completed treatment” predictive models

Supplement 1: Descriptive statistics for categorical variables

Variable	Value	All Sample		# of goals set = 0		# of goals set > 0		# Self Help Groups Attended During Treatment = 0		# Self Help Groups Attended During Treatment > 0	
		Frequency	Percentage	Frequency	Percentage	Frequency	Percentage	Frequency	Percentage	Frequency	Percentage
Race	American Indian or Alaska Native	1	0.4	0	0	1	0.4	0	0	1	0.5
	Bi-racial	4	1.6	1	4.3	3	1.3	1	2.5	3	1.4
	Black or African American	225	87.9	20	87	205	88	36	90	189	87.5
	Latin American/Hispanic	1	0.4	0	0	1	0.4	0	0	1	0.5
	Other	2	0.8	0	0	2	0.9	0	0	2	0.9
	White	23	9	2	8.7	21	9	3	7.5	20	9.3
Marital.Status	Divorced/Seperated	29	11.3	2	8.7	27	11.6	3	7.5	26	12
	Married	8	3.1	0	0	8	3.4	0	0	8	3.7
	Single	218	85.2	21	91.3	197	84.5	37	92.5	181	83.8
	Widow	1	0.4	0	0	1	0.4	0	0	1	0.5
Has.an.established.Support.System.Prior.to.Admissions.	0 (No)	17	6.6	2	8.7	15	6.4	5	12.5	12	5.6
	1 (Yes)	239	93.4	21	91.3	218	93.6	35	87.5	204	94.4
Referral	Aniz, Inc.	1	0.4	0	0	1	0.4	0	0	1	0.5
	Atlanta Public Defender Judicial Circuit	3	1.2	0	0	3	1.3	1	2.5	2	0.9
	Church	1	0.4	0	0	1	0.4	0	0	1	0.5
	Clifton Sanctuary Ministries	1	0.4	0	0	1	0.4	0	0	1	0.5
	DeKalb County Public Defenders Office	9	3.5	0	0	9	3.9	2	5	7	3.2
	Dekalb Crisis Center	109	42.6	13	56.5	96	41.2	19	47.5	90	41.7
	Department of Corrections	4	1.6	0	0	4	1.7	1	2.5	3	1.4
	Dept. of Corrections	1	0.4	0	0	1	0.4	0	0	1	0.5
	Dreamworks	1	0.4	0	0	1	0.4	0	0	1	0.5
	Family	3	1.2	1	4.3	2	0.9	0	0	3	1.4
	Friend	1	0.4	0	0	1	0.4	0	0	1	0.5
	Fulton County Jail	27	10.5	1	4.3	26	11.2	1	2.5	26	12
	Gateway Center	2	0.8	0	0	2	0.9	0	0	2	0.9
	Georgia Regional Hospital	1	0.4	0	0	1	0.4	0	0	1	0.5
	Highland Rivers	1	0.4	0	0	1	0.4	0	0	1	0.5
	McIntosh Trail Community. Service Board	1	0.4	0	0	1	0.4	1	2.5	0	0
	Newport Behaviorial Health	11	4.3	0	0	11	4.7	4	10	7	3.2
	Self	53	20.7	8	34.8	45	19.3	8	20	45	20.8
	St Jude's Recovery Center	24	9.4	0	0	24	10.3	2	5	22	10.2
	Stand Alumni	1	0.4	0	0	1	0.4	0	0	1	0.5
	Willowbrooke at Tanner	1	0.4	0	0	1	0.4	1	2.5	0	0
Court.Ordered.to.Participate.	0 (No)	206	80.5	21	91.3	185	79.4	36	90	170	78.7
	1 (Yes)	50	19.5	2	8.7	48	20.6	4	10	46	21.3
Discharge.Type	0 (Not compmpleted succcessfully)	141	55.1	19	82.6	122	52.4	36	90	105	48.6
	1 (Completed successfully)	115	44.9	4	17.4	111	47.6	4	10	111	51.4
Drug.of.Choice	Alcohol	97	37.9	9	39.1	88	37.8	15	37.5	82	38
	Amphetamine	9	3.5	1	4.3	8	3.4	0	0	9	4.2
	Cannabis	22	8.6	2	8.7	20	8.6	6	15	16	7.4
	Cocaine	111	43.4	9	39.1	102	43.8	18	45	93	43.1
	Heroin	1	0.4	0	0	1	0.4	0	0	1	0.5
	Opioids	14	5.5	1	4.3	13	5.6	1	2.5	13	6
	Unknown	2	0.8	1	4.3	1	0.4	0	0	2	0.9
Secondary.Drug.of.Choice	Alcohol	46	18	1	4.3	45	19.3	7	17.5	39	18.1
	Amphetamine	9	3.5	0	0	9	3.9	3	7.5	6	2.8
	Cannabis	40	15.6	3	13	37	15.9	4	10	36	16.7
	Cocaine	43	16.8	1	4.3	42	18	6	15	37	17.1
	N/A	86	33.6	15	65.2	71	30.5	11	27.5	75	34.7
	Opiods	1	0.4	0	0	1	0.4	0	0	1	0.5
	Opioids	7	2.7	1	4.3	6	2.6	0	0	7	3.2
	Polysubstance	4	1.6	0	0	4	1.7	1	2.5	3	1.4
	Unknown	20	7.8	2	8.7	18	7.7	8	20	12	5.6

		All Sample		# of goals set = 0		# of goals set > 0		# Self Help Groups Attended During Treatment = 0		# Self Help Groups Attended During Treatment > 0	
**Variable**	**Value**	**Frequency**	**Percentage**	**Frequency**	**Percentage**	**Frequency**	**Percentage**	**Frequency**	**Percentage**	**Frequency**	**Percentage**
**Intravenous.User**	**0 (No)**	240	93.8	22	95.7	218	93.6	38	95	202	93.5
	**1 (Yes)**	16	6.2	1	4.3	15	6.4	2	5	14	6.5
**Dual.Diagnosis**	**0 (No)**	184	71.9	18	78.3	166	71.2	29	72.5	155	71.8
	**1 (Yes)**	72	28.1	5	21.7	67	28.8	11	27.5	61	28.2
**Principal.Mental.Health.Diagnosis**	**0 (No)**	194	75.8	19	82.6	175	75.1	27	67.5	167	77.3
	**1 (Yes)**	62	24.2	4	17.4	58	24.9	13	32.5	49	22.7
**Secondary.Mental.Health.Diagnosis**	**0 (No)**	241	94.1	22	95.7	219	94	34	85	207	95.8
	**1 (Yes)**	15	5.9	1	4.3	14	6	6	15	9	4.2
**Prescribed.Medication**	**0 (No)**	160	62.5	14	60.9	146	62.7	21	52.5	139	64.4
	**1 (Yes)**	96	37.5	9	39.1	87	37.3	19	47.5	77	35.6
**Funding**	**Beacon Health**	230	89.8	20	87	210	90.1	36	90	194	89.8
	**Medicaid**	24	9.4	2	8.7	22	9.4	4	10	20	9.3
	**None**	1	0.4	0	0	1	0.4	0	0	1	0.5
	**Unknown**	1	0.4	1	4.3	0	0	0	0	1	0.5
**Homeless.In.Shelter.**	**0 (No)**	244	95.3	22	95.7	222	95.3	39	97.5	205	94.9
	**1 (Yes)**	12	4.7	1	4.3	11	4.7	1	2.5	11	5.1
**Homeless.Out.of.Shelter.**	**0 (No)**	82	32	10	43.5	72	30.9	17	42.5	65	30.1
	**1 (Yes)**	174	68	13	56.5	161	69.1	23	57.5	151	69.9
**Entered.into.STAND.s.Housing.Program**	**0 (No)**	27	10.5	5	21.7	22	9.4	7	17.5	20	9.3
	**1 (Yes)**	229	89.5	18	78.3	211	90.6	33	82.5	196	90.7
**Disrupted.Housing.**	**0 (No)**	241	94.1	21	91.3	220	94.4	37	92.5	204	94.4
	**1 (Yes)**	15	5.9	2	8.7	13	5.6	3	7.5	12	5.6
**Highest.Grade.Completed**	**7**	3	1.2	1	4.3	2	0.9			3	1.4
	**8**	6	2.3	0	0	6	2.6			6	2.8
	**9**	8	3.1	2	8.7	6	2.6	2	5	6	2.8
	**10**	19	7.4	4	17.4	15	6.4	5	12.5	14	6.5
	**11**	29	11.3	2	8.7	27	11.6	7	17.5	22	10.2
	**12**	110	43	8	34.8	102	43.8	16	40	94	43.5
	**13**	2	0.8	0	0	2	0.9	1	2.5	1	0.5
	**14**	69	27	5	21.7	64	27.5	9	22.5	60	27.8
	**16**	8	3.1	1	4.3	7	3			8	3.7
	**18**	2	0.8	0	0	2	0.9			2	0.9
**Has.Dependent.children**	**0 (No)**	192	75	20	87	172	73.8	31	77.5	161	74.5
	**1 (Yes)**	64	25	3	13	61	26.2	9	22.5	55	25.5
**Employed.Prior.to.Admissions**	**0 (No)**	238	93	21	91.3	217	93.1	38	95	200	92.6
	**1 (Yes)**	18	7	2	8.7	16	6.9	2	5	16	7.4
**Access.to.Insurance.at.Admissions**	**0 (No)**	224	87.5	23	100	201	86.3	37	92.5	187	86.6
	**1 (Yes)**	32	12.5	0	0	32	13.7	3	7.5	29	13.4
**Criminal.Justice.System.Involvement.at.Admissions**	**0 (No)**	134	52.3	14	60.9	120	51.5	25	62.5	109	50.5
	**1 (Yes)**	122	47.7	9	39.1	113	48.5	15	37.5	107	49.5
**X.Previous.Psychiatric.Inpatient..Crisis.Unit.Stays.Prior.to.Admisisons**	**0 (No)**	88	34.4	7	30.4	81	34.8	7	17.5	81	37.5
	**1 (Yes)**	168	65.6	16	69.6	152	65.2	33	82.5	135	62.5
**Reduced.Utilization.of.Psychiatric.Inpatient..Crisis.Stabilization.Units**	**0 (No)**	82	32	6	26.1	76	32.6	7	17.5	75	34.7
	**1 (Yes)**	174	68	17	73.9	157	67.4	33	82.5	141	65.3
**Receiving.SSI.at.Intake.**	**0 (No)**	232	90.6	21	91.3	211	90.6	36	90	196	90.7
	**1 (Yes)**	24	9.4	2	8.7	22	9.4	4	10	20	9.3
**Receving.SNAP.s.at.Intake.**	**0 (No)**	198	77.3	18	78.3	180	77.3	30	75	168	77.8
	**1 (Yes)**	58	22.7	5	21.7	53	22.7	10	25	48	22.2
**Linked.to.SNAP.s.after.Intake.**	**0 (No)**	72	28.1	14	60.9	58	24.9	20	50	52	24.1
	**1 (Yes)**	184	71.9	9	39.1	175	75.1	20	50	164	75.9
**Already.had.a.Primary.Care.Physician.at.Intake**	**0 (No)**	200	78.1	18	78.3	182	78.1	31	77.5	169	78.2
	**1 (Yes)**	56	21.9	5	21.7	51	21.9	9	22.5	47	21.8
**Linked.to.a.Primary.Care.Physician.During.Treatment**	**0 (No)**	81	31.6	12	52.2	69	29.6	25	62.5	56	25.9
	**1 (Yes)**	175	68.4	11	47.8	164	70.4	15	37.5	160	74.1

Variable	Value	All Sample Frequency	All Sample Percentage	# of goals set = 0 Frequency	# of goals set = 0 Percentage	# of goals set > 0 Frequency	# of goals set > 0 Percentage	# Self Help Groups Attended During Treatment = 0 Frequency	# Self Help Groups Attended During Treatment = 0 Percentage	# Self Help Groups Attended During Treatment > 0 Frequency	# Self Help Groups Attended During Treatment > 0 Percentage
**Has.a.Chronic.Medical.Issues.**	**0 (No)**	98	38.3	5	21.7	93	39.9	17	42.5	81	37.5
	**1 (Yes)**	158	61.7	18	78.3	140	60.1	23	57.5	135	62.5
**Access.to.Transportation.at.Intake.**	**0 (No)**	236	92.2	21	91.3	215	92.3	36	90	200	92.6
	**1 (Yes)**	20	7.8	2	8.7	18	7.7	4	10	16	7.4
**Linked.to.Transportation.Services.**	**0 (No)**	17	6.6	2	8.7	15	6.4	8	20	9	4.2
	**1 (Yes)**	239	93.4	21	91.3	218	93.6	32	80	207	95.8
**Days.In.Program..to.Discharge.**	**0 (<=90)**	108	42.2	15	65.2	93	39.9	31	77.5	77	35.6
	**1 (>90)**	148	57.8	8	34.8	140	60.1	9	22.5	139	64.4
**Age_Approximation**	**21-25**	1	0.4	0	0	1	0.4	0	0	1	0.5
	**26-30**	10	3.9	3	13	7	3	2	5	8	3.7
	**31-35**	24	9.4	2	8.7	22	9.4	6	15	18	8.3
	**36-40**	24	9.4	3	13	21	9	6	15	18	8.3
	**41-45**	40	15.6	6	26.1	34	14.6	5	12.5	35	16.2
	**46-50**	34	13.3	2	8.7	32	13.7	7	17.5	27	12.5
	**51-55**	64	25	3	13	61	26.2	8	20	56	25.9
	**56-60**	43	16.8	4	17.4	39	16.7	6	15	37	17.1
	**61-65**	16	6.2	0	0	16	6.9	0	0	16	7.4
**Court.Prescribed.Medication**	**0 (No)**	160	62.5	14	60.9	146	62.7	21	52.5	139	64.4
	**1 (Yes)**	96	37.5	9	39.1	87	37.3	19	47.5	77	35.6
**Criminal.Justice.System.Involvement.at.Admissions.**	**0 (No)**	134	52.3	14	60.9	120	51.5	25	62.5	109	50.5
	**1 (Yes)**	122	47.7	9	39.1	113	48.5	15	37.5	107	49.5
**Reduced.Util.of.Psychiatric.Inpatient..Crisis.Stabilization.Units.**	**0 (No)**	82	32	6	26.1	76	32.6	7	17.5	75	34.7
	**1 (Yes)**	174	68	17	73.9	157	67.4	33	82.5	141	65.3

Supplement 2: Descriptive statistics for continuous variables

	All Sample			
Variable	Min	Max	Mean	Median
X..of.Previous.Treatment.Episodes	0	10	2.078125	2
X..of.GOALS.Set.by.Client	0	11	3.33203125	3
X..of.Self.Help.Groups.Attended.within.the.30.Days.Prior.to.Admissions	0	30	1.33984375	0
X..of.Self.Help.Groups.Attended.During.Treatment	0	80	14.5546875	10

	# of goals set = 0				# of goals set > 0			
	Min	Max	Mean	Median	Min	Max	Mean	Median
X..of.Previous.Treatment.Episodes	0	6	1.826086957	2	0	10	2.103004292	2
X..of.GOALS.Set.by.Client	0	0	0	0	1	11	3.660944206	3
X..of.Self.Help.Groups.Attended.within.the.30.Days.Prior.to.Admissions	0	12	1	0	0	30	1.373390558	0
X..of.Self.Help.Groups.Attended.During.Treatment	0	80	12.67391304	5	0	80	14.74034335	10

	# Self Help Groups Attended During Treatment = 0				# Self Help Groups Attended During Treatment > 0			
	Min	Max	Mean	Median	Min	Max	Mean	Median
X..of.Previous.Treatment.Episodes	0	10	2	1.5	0	10	2.092592593	2
X..of.GOALS.Set.by.Client	0	8	2.325	2	0	11	3.518518519	3
X..of.Self.Help.Groups.Attended.within.the.30.Days.Prior.to.Admissions	0	20	0.825	0	0	30	1.435185185	0
X..of.Self.Help.Groups.Attended.During.Treatment	0	0	0	0	1	80	17.25	10

Supplement 3: Virtual twins stage 1 accuracies and AUCs for “>90 days” predictive models

	Logistic Regression	Gradient Boosting Machine	Random Forest	Deep Learning
**AUC**	0.772722925	0.767088328	0.781835478	0.748033891
**Accuracy**	0.750649351	0.731168831	0.746753247	0.74025974

Supplement 4: Virtual twins stage 1 accuracies and AUCs for "successfully completed treatment" predictive models

	Logistic Regression	Gradient Boosting Machine	Random Forest	Deep Learning
**AUC**	0.857546338	0.881673345	0.886462079	0.84119664
**Accuracy**	0.818181818	0.831168831	0.831168831	0.797402597
